# Reconstruction of pulse noisy images via stochastic resonance

**DOI:** 10.1038/srep10616

**Published:** 2015-06-12

**Authors:** Jing Han, Hongjun Liu, Qibing Sun, Nan Huang

**Affiliations:** 1 State Key Laboratory of Transient Optics and Photonics, Xi’an Institute of Optics and Precision Mechanics, Xi’an, 710119, China

## Abstract

We investigate a practical technology for reconstructing nanosecond pulse noisy images via stochastic resonance, which is based on the modulation instability. A theoretical model of this method for optical pulse signal is built to effectively recover the pulse image. The nanosecond noise-hidden images grow at the expense of noise during the stochastic resonance process in a photorefractive medium. The properties of output images are mainly determined by the input signal-to-noise intensity ratio, the applied voltage across the medium, and the correlation length of noise background. A high cross-correlation gain is obtained by optimizing these parameters. This provides a potential method for detecting low-level or hidden pulse images in various imaging applications.

In many imaging fields, a low-level signal is often merged by noise and difficult to be distinguished. In linear systems, the noise is considered harmful to signal. While in nonlinear systems, an appropriate level of noise is benefit to recover signal because of the more complex dynamics, which is described as “stochastic resonance”^1^. The signal can be recovered and amplified from a low signal-to-noise intensity ratio, which is not achieved in conventional linear systems. In addition, this effect is also applied to the signals hidden by the noise with approximate frequency. Many works of stochastic resonance have been reported from climatic patterns^2^ to electrical systems^3^ and biology^4^. Most of them focus on the one-dimensional beams rather than the two-dimensional images and are restricted to a threshold^1^. However, no feedback or threshold is required in the stochastic resonance based on modulation instability^3^,^5^,^6^. D. V. Dylov *et al.* have demonstrated the effect using continuous wave with spatially coherent-incoherent coupling^7^. Their results suggest a general method of reconstructing images by seeding instability in nonlinear imaging systems. While, the nanosecond pulse images is widely used in optical processing^8^,^9^,^10^. Especially, in remote light detection and transmission, the nanosecond pulse images are usual low-level and relatively low repetition frequency. These characteristics effectively reduce the power required and the loss in long distance, but also bring new influences to reconstruct the signal. We must consider the impacts of pulse width and repetition frequency on the dynamic process of modulation instability. To provide a theoretical guidance for reconstructing a nanosecond pulse signal, it is necessary to build a theoretical model of modulation instability suitable for the pulse signal.

In this paper, we have established a theoretical model of stochastic resonance for optical nanosecond signal to recover the pulse noise-hidden image. The reconstruction of pulse images is achieved when the energy transfers to signal from noise background. The instability of this effect is generated in coherent-incoherent coupling related to the properties of input pulse. According to this model, the impacts of parameters on stochastic resonance are analyzed in detail.

## Results

### Schematic diagram and design

**Figure 1:**
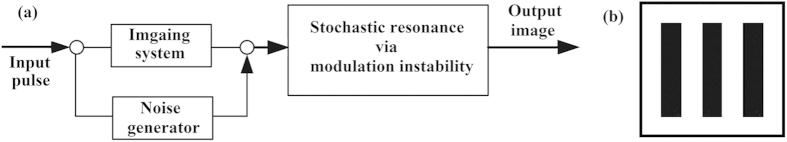
(**a**) Schematic diagram to reconstruct noise-hidden images of nanosecond pulse via stochastic resonance.(**b**) The original pure image carried by signal beam.

Figure 1(a) details the schematic diagram of the stochastic resonance system based on modulation instability. In simulation, the wavelength of input nanosecond pulse is 532 nm with the repetition frequency 10 kHz. This pulse is divided into two beams with an adjustable splitting ratio. The signal beam is a coherent pulse carrying an image of resolution chart as Fig. 1(b). The noise beam is a spatially incoherent pulse with random phase fluctuations. A “Lens-Diffuser-Lens” section in noise generator determines the correlation length of the incoherent beam^11^. The stochastic resonance is generated when the two beams are simultaneously and collinearly injected into a photorefractive crystal. We use the parameters of SBN:60:Cr due to its characteristics of fast response time 0.5 ms and high electro-optic coefficient 1340 pm/V. The nonlinear coefficient is controlled by tuning an applied electric field parallel to the optical axis.

### Pulse output images and cross-correlation

To evaluate the impacts of parameters on the stochastic resonance according to the model, we use the cross-correlation gain to quantitative measure the improvement of the similarity between input images and output images. The normalized cross-correlation coefficient between the pure resolution chart and the pulse images is defined as reference^12^.

**Figure 2:**
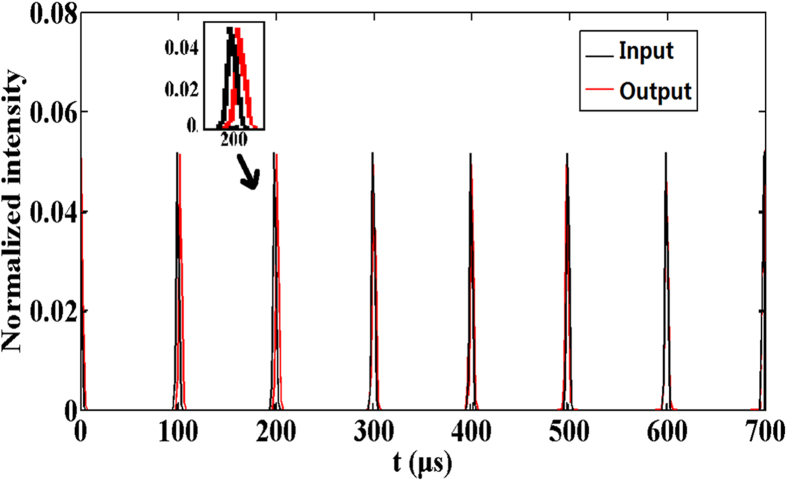
The time profiles of the input and output pulse. The normalized value of the input image is set at 0.05.

The profiles of input and output pulse in time domain are shown in Fig. 2. It is clearly seen that in the first few cycles, the output waveform is delayed relative to the input pulse, in which the back edge has a tail as the illustration shows. With the increase of propagation time, the delay decreases, thus the profiles are approximately the same in time domain. The reason is that the response time of SBN:60:Cr is 0.5 ms much longer than the period of nanosecond pulse. To fully couple in the photorefractive crystal, it needs to absorb enough photons to trigger carriers, which are necessary to establish the spatial coupling electric field. This process takes more time when the intensity of input pulse is weak. When the propagation time in crystal is enough to stabilize the extreme values of *E*_*sc*_, the tail of output pulse profile will vanish. A photorefractive medium of fast response time can accelerate this process. However, we should consider both of the electric-optic coefficient and the response time when selecting the suitable photorefractive crystal.

**Figure 3:**
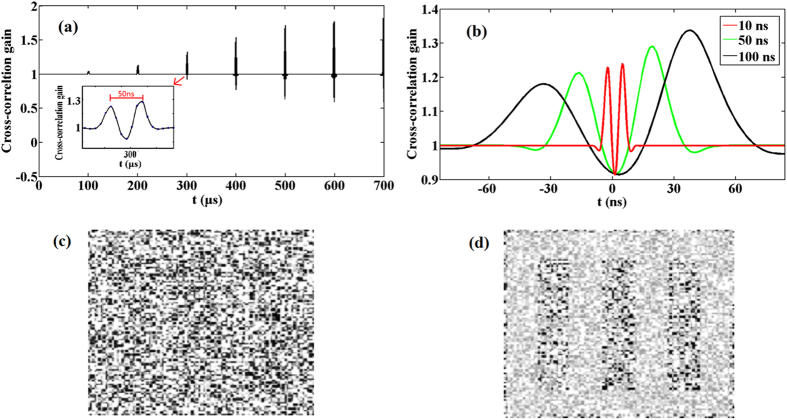
(**a**) Cross-correlation gain varies with propagation time. (**b**) Cross-correlation gain of different input pulse width 10 ns, 50 ns, 100 ns, respectively. (**c**) The input pulse image and (**d**) output image. Other parameters normalized noise intensity *D* = 30, *E*_0_ = 5100 V/m, *l*_*c*_ = 97 μm.

Meanwhile, the response time of photorefractive medium also has influence on the cross-correlation gain as depicted from Fig. 3(a). The extreme values of the curve vary with the propagation time. Nevertheless, they become almost equal after 500 μs. The pulse width in Fig. 3(a) is 50 ns (full width of half maximum, FWHM). Within one pulse cycle as shown in the illustration, the cross-correlation gain of output images has two unequal peaks. Furthermore, it decreases to a minimum at the peak of the input pulse. The reason is that the coherency of input image decreases at this time because the confusion of neighboring hot spots exacerbates as the increase of intensity^13^. Therefore, the corresponding values of the curve are less than 1, which means that the quality of the output pulse images is worse than input. Contrarily, the others values larger than 1 are benefit to recover the signal images. In the photorefractive crystal, the nonlinear coefficient *γ* is dynamical because of the fluctuant *E*_*sc*_. The density of electrons simulated by the input pulse is periodically modulated. The effect of stochastic resonance is generated as the reach of input pulse. It submerges when the intensity of the input pulse begins to decline since there is no driving gradient anymore. The spatial coupling electric field *E*_*sc*_ is established with a form of oscillation^14^. An effectively recovered image is obtained as Fig. 3(d), when the extreme values of *E*_*sc*_ begin to be equilibrium after some distance of propagation. In addition, the influence of pulse width on the cross-correlation gain is shown in Fig. 3(b). These curves center around 300 μs as the illustration in Fig. 3(a). It shows that the pulse width changes the location of the peaks of cross-correlation gain. The reason is that the pulse width influences the time phase of writing and erasing of the spatial coupling electric field. While the maximal complex amplitude of the spatial coupling electric field is limited to *mE*_0_ under the condition of unsaturation. The degree of modulation *m* is related to the change of the intensity of input pulse rather than the intensity itself. A weak pulse can also leads to the considerable nonlinearity in the photorefractive medium. The intensity *I* only changes the rate to generate the effect of photorefractive. The peaks in the cross-correlation gain curves with different pulse widths will be equal gradually. This means that the pulse width does not impact the optimal value of cross-correlation gain. Meanwhile, the effect of stochastic resonance for reconstructing the noisy images also applies to the input images of several nanosecond pulse width.

**Figure 4:**
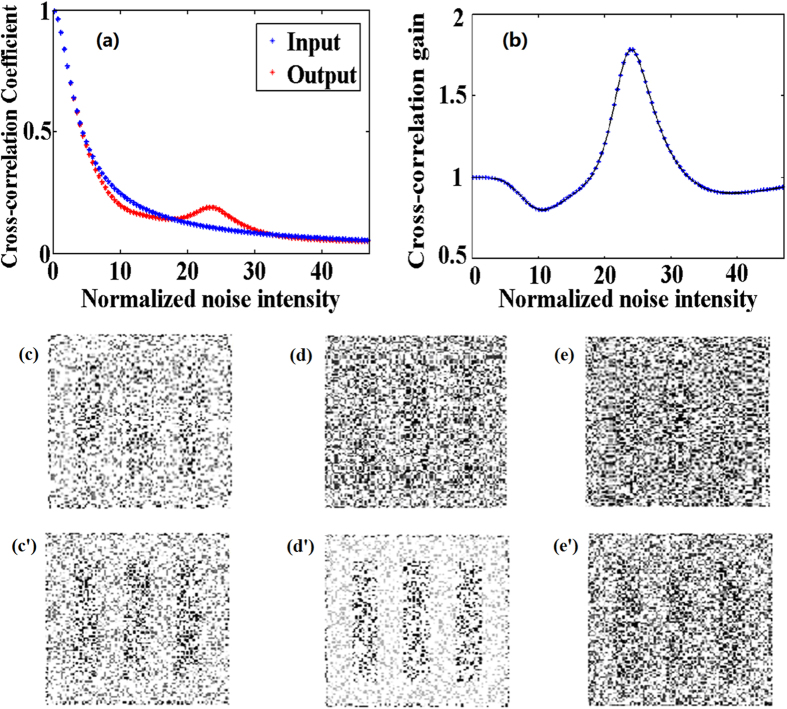
(**a**) The cross-correlation coefficient and (**b**) cross-correlation gain versus *D* with *E*_0_ = 5100 V/m, *l*_*c*_ = 97 μm, the pulse width is fixed at 50 ns.(**c**)–(**e**) The pulse input images for D = 20, 25 and 30, respectively. (**c’**)–(**e’**) are the corresponding output images to (**c**)–(**e**), respectively.

According to the theoretical model, the cross-correlation coefficient and gain as functions of normalized noise intensity *D* from 0 to 50 are numerically calculated as shown in Fig. 4(a,b), respectively. As clearly seen from Fig. 4(a), the cross-correlation coefficient of input pulse images decreases with the increase of noise intensity. While this coefficient of output images has a turning when *D* is located between 20 and 30. The corresponding cross-correlation gain is larger than 1 as shown in Fig. 4(b). An optimal value of 1.7 is obtained as Fig. 4(d’) with the normalized input noise intensity *D* = 25. Figure 4(c,e’) depicts the ability to reconstruct noisy images via stochastic resonance with different *D*. An appropriate intensity of disordered noise is necessary to the energy coupling and the maximal growth of signal energy. When *D* is too small, the energy coupled into the signal is less compared to the original noise intensity, thus the cross-correlation gain is not apparently improved. As the intensity of noise increases, the modulations become more pronounced with higher visibility. In other words, the fastest-growing modes corresponding to the more efficient energy transfer needs higher momenta. While a too large *D* will dominate this system and destroy the conditions of stochastic resonance, which leads to the distortion of output images. This signature of stochastic resonance for nanosecond pulse images is caused by the instability of energy coupling between weak signals and random noise^1^,^7^. Therefore, the noise intensity should be carefully designed to reconstruct the pulse noisy images with a high cross-correlation gain.

**Figure 5:**
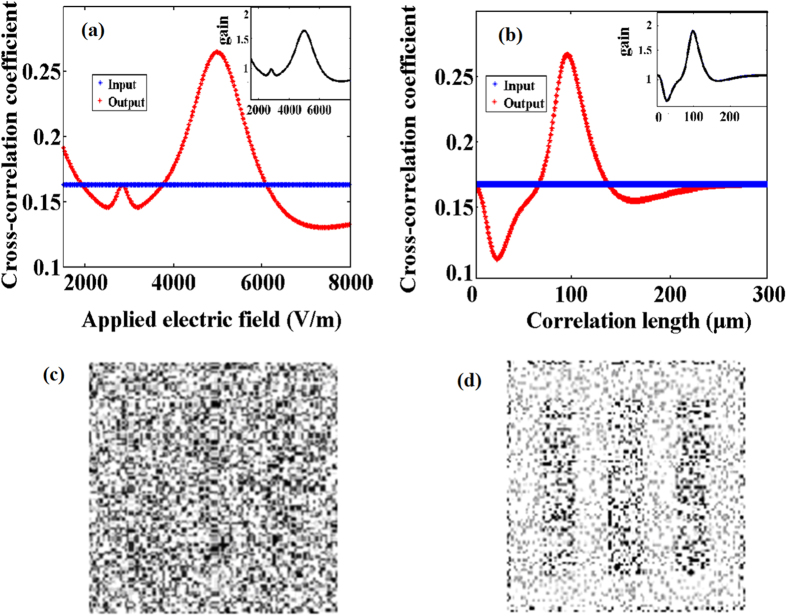
The cross-correlation coefficient and gain (**a**) The output versus the applied electric field *E*_0_  with *l*_c_ = 97 μm.(**b**) The output versus the correlation length *l*_c_ with *E*_0_  = 5100 V/m. (**c**) The input pulse image and (**d**) output pulse image optimized by these parameters. D = 25.

Additionally, the impacts of the applied electric field *E*_0_ and the correlation length on the stochastic resonance are numerically calculated as shown in Fig. 5(a,b), respectively. It is found that there exists an electric field of 5100 V/m and a correlation length of 97 μm to optimize the cross-correlation gain with the output Fig. 5(d). The influence of *E*_0_ on the nonlinear coefficient mainly depends on changing the refraction index *n*, which controls the strength of the nonlinear coupling. The quality of output pulse image improves until a maximal cross-correlation gain of 1.7 is reached, beyond which the quality of output pulse images degrades. This dynamical process can be explained with the theory of turbulence. When Δ*n* increases with the voltage, it triggers the modulation instability to drive the transfer of energy. The distribution of total energy of the coherent signal and incoherent noise is reconstructed due to the movement of particles because the Δ*n* keeps acting on the interface. The efficiency of energy transferring from high-level noise to low-level signal reaches a maximum at an optimal applied voltage. At a higher Δ*n*, the turbulent dynamics continues mixing signal and noise, but the border of signal and noise will degrade in coherent-incoherent snake-like instability^15^. The cross-correlation gain reflects this property of stochastic resonance versus the applied voltage.

Moreover, when the total intensity of input pulse, the separation ratio of two beams and the applied voltage are kept constant, the dynamical coupling is determined by the correlation length, which can be changed by varying the properties of the lens. This parameter mainly depends on the average speckle size, which indicates the overlap degree of the spectral peaks of input beam in the momentum-space. For a single-humped distribution, the clear dynamics requires strong nonlinearity. When the spectral peaks suitably overlap giving an unstable condition, the exchange of momentum is most efficient to provide a significant nonlinearity. A large *l*_*c*_ indicates that the input beam is too coherent, so that only few disordered particles of noise are in resonance with the signal wave. While if the input beam is too incoherent with a small *l*_*c*_, the transfer of spectral energy is difficult to chance, which means that exceeds the ability of energy coupling in the stochastic resonance. It is feasibility to tune the correlation length to acquire an optimal cross-correlation gain of output pulse image.

## Discussion.

Diffraction and nonlinearity are the two necessary conditions to generate modulation instability in a nonlinear system^16^. They determine the intensity of incident wave enhanced or diminished in propagation. The specific process is positive exchange during propagation. The initial weak signals generate a potential well, which is reinforced by the nonlinear coupling process^7^. This coupling is necessary to cause the energy transfer between different components. However, the potential well is unsteady due to the chaotic particles of partially coherent pulse. For the incident pulse, depth of this potential well varies with its instantaneous intensity and the existence time of potential well is influenced by its pulse width. Therefore, the instable energy transfer of noise to the signal varies with the pulse phase, resulting that the cross-correlation gain is subject to the relevant noise intensity, pulse width and repetition frequency of the incident pulse. The applied electric field and other factors have effects on this coupling process. Tuning parameters suitably can contribute the energy transferring from the high-level noise to the low-level signal. The output signal-to-noise ratio reaches a peak, which reflects the phenomena of stochastic resonance.

In conclusion, we report an effective technology to reconstruct the noisy images of nanosecond pulse. Meanwhile, a theoretical model of stochastic resonance for the pulse signals is built to obtain an optimal cross-correlation gain of output images. We also investigate the influences of the input noisy intensity, the applied voltage, and the correlation length on stochastic instability, respectively. The input pulse images hidden by noise might still possess necessary correlations about the initial information, which can be recovered by carefully designing parameters. This provides a potential method to recover the original pulse images from noise-hidden images in various applications of pulse imaging process.

## Method

### The theory of stochastic resonance for optical pulse noisy signal

The theoretical model is based on the nonlinear Schrödinger (NLS) equation including temporal terms, which can be obtained from the Maxwell equations^17^. Under the condition that the envelope of nanosecond pulses slowly varies^18^, the packet of coherent wave *ψ*(***r***, *z*, *t*) is expressed as
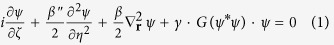
where ζ = *z* and η = *t* – *z*/*ν*_*g*_ is the coordinates system, *ν*_*g*_ = (*dk*/*dω*|_*ωl*_)^−1^ is the group velocity, *z* is the propagation distance, ***r*** is the spatial dispersive variable, *t* is the temporal phase, *γ* is the nonlinear coefficient of spatial coupling electric field, and *β* is the diffraction coefficient. *G*(ψ*ψ) is the statistical nonlinear response function of the medium, which is also valid even that the response time of medium is much longer than the characteristic time of the statistical wave packet fluctuations.

Considering the dynamics of the low-level signals, we take the instability-linear perturbation theory to focus on the response of the noise to the driving signal. In the geometrical optics, the approximation Δ***k***·Δ***r*** ≫ 2*π* is obviously applied to the nanosecond pulse according to references^19^,^20^,^21^. Neglecting the linear loss and the high-order nonlinear contribution^7^,^20^,^21^, Equation (1) can be evolved to
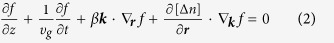
where *f* is the distribution of pulse intensity *I*, Δ*n* is the change of refractive index due to nonlinearity. With the distribution of spatially uniform and temporal periodicity, the solution *f*(***r***, ***k***, *z*, *t*) is descried as
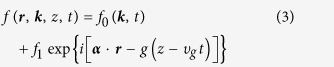
where *f*_0_ is the homogeneous term for incident pulse light, *f*_1_ is the perturbation term with wavenumber ***α***, and *g* is the growth rate. The evolution of input pulse is not only related to the propagation distance and transverse coordinates, but the temporal phase. Moreover, the space phase can be separated from the temporal phase^18^. The first term in Equation (3) is rewritten as *f*_0_(***k***, *t*) = *f*_0_(***k***)·*δ*(*t*), where *δ*(*t*) = exp[–2ln2·(*t* + *nT*)^2^/*τ*^2^] indicates the temporal distribution of nanosecond pulse, *T* is the period gap of pulse and *τ* is the pulse width. Hence, Equation (3) can be rewritten as
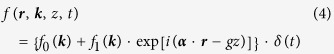


For the low-level intensity of input pulse images, we can linearize the perturbation to treat each pixel individually. According to Equation (2)–(4), 3, 4, the general solution for *g* can be obtained using the method of Laplace transform and Taylor series^22^. It is described as
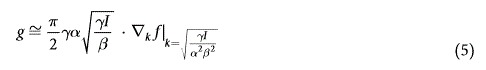


In Equation (5), the nonlinear coefficient *γ* in photorefractive crystal is determined by the spatial coupling electric field *E*_*sc*_, which is given by^14^
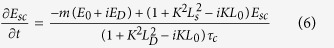


The initial conditions is *E*_*sc*_ = 0. In Equation (6), the degree of modulation *m* is the ratio of complex amplitude of the perturbation term *f*_1_ to the homogeneous term *f*_0_; *E*_*0*_ is the voltage applied across the optical axis of the photorefractive crystal; *E*_*D*_ is the electric field of diffusion; *E*_*S*_ is the electric field of maximum space charge; *K* is the repetition frequency of input pulse; *τ*_*c*_ is the response time of the crystal; *L*_0_, *L*_*s*_ and *l*_0_ are lengths of drift, Debye shielding and traction of external electric field, respectively. The nonlinear coefficient satisfies *γ* = *n*_0_^3^*r*_*eff*_*E*_*sc*_cos*θ*, in which *r*_*eff*_ is the relevant electro-optic coefficient, *n*_*0*_ is the ordinary refractive index, and *θ* is the incident angle.

Meanwhile, the correlation length *l*_c_ in Equation (5) indicates the variance of intensity of input noise, which effects on the efficiency of energy coupling. It is controlled by the focal length of lens and roughness of diffuser, which is used to generate the spatial incoherent pulse^11^,^23^
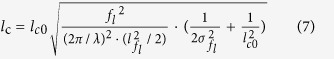
where *l*_c0_ = 0.707*λf*_*l*_ /*πa*, *f*_*l*_ is the focal length of lens, *a* is a constant related to the roughness of diffuser, *λ* is the wavelength and *l*_*fl*_ is determined by the correlation length of the laser source.

According to Equation (5)–(7), 6, 7, when *f*_0_(***k***) satisfies the Gaussian distribution
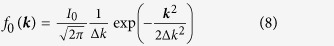
the growth rate *g* is given by
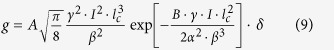
in which *A* and *B* are effective mode-dependent normalization constants giving the height and location of the visibility peak^7^.

## Additional Information

**How to cite this article**: Han, J. *et al.* Reconstruction of pulse noisy images via stochastic resonance. *Sci. Rep.* **5**, 10616; doi: 10.1038/srep10616 (2015).
